# The WRKY Transcription Factor OsWRKY54 Is Involved in Salt Tolerance in Rice

**DOI:** 10.3390/ijms231911999

**Published:** 2022-10-09

**Authors:** Jingjing Huang, Fuhang Liu, Dong Chao, Boning Xin, Kui Liu, Shuling Cao, Xingxiang Chen, Liyun Peng, Baolei Zhang, Shan Fu, Jixing Xia

**Affiliations:** State Key Laboratory for Conservation and Utilization of Subtropical Agro-Bioresources, College of Life Science and Technology, Guangxi University, Nanning 530004, China

**Keywords:** salinity, rice, WRKY, transcription factor, RNA-seq

## Abstract

Salt stress is a critical limiting factor for rice growth and production. Although numerous salt-tolerant genes have been identified, the mechanism underlying salt stress tolerance in rice remains unclear. This study reports the need for an uncharacterized WRKY transcription factor OsWRKY54 for rice salt-tolerance. Salt stress resulted in a rapid induction of *OsWRKY54* expression in roots. Immunostaining analysis showed that it was mainly expressed in the stele. The loss of *OsWRKY54* resulted in greater Na accumulation in shoots and enhanced sensitivity of rice plants to salt stress. The real-time quantitative PCR (qRT-PCR) and transcriptome analysis revealed that OsWRKY54 regulated the expression of some essential genes related to salt tolerance, such as *OsNHX4* and *OsHKT1;5.* Furthermore, OsWRKY54 was found to regulate *OsHKT1;5* expression by directly binding to the W-box motif in its promoter. Thus, these results indicated that *OsWRKY54* was a critical regulatory factor in salt tolerance in rice.

## 1. Introduction

Soil salinity is a globally significant environmental issue for agricultural productivity. Approximately 20% of the globally cultivated land and 50% of the total irrigated land are severely affected by high salt stress [1]. High sodium (Na^+^) concentrations in soil cause osmotic and ionic stresses in plants [2]. Osmotic stress can lead to the inhibition of nutrient balance, cell expansion, and cell division. In contrast, ionic stress is known to cause high Na^+^ accumulation in the shoots, which inhibits cellular metabolism, including enzyme activity, protein synthesis, and photosynthesis [2,3]. Thus, plants have developed a series of strategies to cope with the detrimental effects of salt stress in order to adapt to adverse environments, such as the extrusion of Na^+^ from the roots and leaf tissue and the sequestration of Na^+^ into vacuoles.

Many genes and multiple mechanisms control plant responses to abiotic stresses. As critical factors, transcription factors (TFs) such as zinc finger protein (bZIP) and the NAC, MYB, and WRKY families are required for plant adaptation to abiotic stress by modulating the expression of their downstream genes [4]. The WRKY TFs family has garnered significant attention for its role in response to abiotic stresses, such as drought, salt, and heat stresses, resulting in the identification of more than 100 in rice and 74 in Arabidopsis WRKY proteins. Typically, WRKY TFs have the highly conserved sequence WRKYGQK at the N-terminal region and contain the zinc finger motif (CX7CX23HXC or CX4-5CX22-23HXH) at the C-terminal region [5,6]. WRKY transcription factors can be divided into three groups (I, II, and III) on the basis of the structure of zinc finger motif and the number of WRKY domains [7]. WRKY transcription factors have the potential to activate or repress the expression of the downstream genes by specifically binding to the W-box motif (TTGACC/T) within their promoters [8].

WRKY TFs play essential roles in response to salt stress in various plants. For example, AtWRKY8 is known to positively regulate salinity stress tolerance in Arabidopsis [9]. The overexpression of AtWRKY74 significantly improves the tolerance of Arabidopsis to salt stress. In tomatoes (*Solanum lycopersicum*), *SlWRKY8* overexpression exhibited an enhanced tolerance of plants to drought and salt stresses [10]. The ectopic overexpression of *ZmWRKY17* decreased Arabidopsis plants’ tolerance to salt stress. Some ABA-response genes in the overexpression lines had a lower expression level compared with the wild type under salt conditions, indicating that ZmWRKY17 might function as a negative regulator involved in salt-stress responses via the ABA signal transduction pathway [11]. In wheat (*Triticum aestivum*), the expression of *TaWRKY75-A* was strongly induced by salt and polyethylene glycol (PEG) treatments. The heterologous expression of *TaWRKY75-A* in Arabidopsis showed the increased resistance of the plants to salt and drought stresses in transgenic plants, indicating that TaWRKY75-A might be involved in salt and drought resistance in wheat [12]. In rice, OsWRKY50 was proposed to positively regulate salt stress response through an ABA-independent signaling pathway [13]. A recent study demonstrated that *OsWRKY87* induced rice salt and drought tolerance by directly regulating the expression of OsABF1 [14]. However, the role of WRKY TFs in regulating rice salt-tolerance remains unclear.

Previous transcriptomic analysis showed that salt treatment resulted in the significant upregulation of a rice salt-responsive gene (LOC_Os05g40080) encoding a WRKY transcription factor OsWRKY54 in the roots [15], suggesting its probable involvement in rice salt-stress response. However, its exact role in rice salt-tolerance was not investigated. In this study, we examined the expression pattern and localization of OsWRKY54 and the physiological phenotypes of its mutant lines under salt treatment. Additionally, the root transcriptomic analysis of *OsWRKY54*-knockout and wild-type plants was performed under normal and high-salt conditions. Our results revealed that OsWRKY54 mediated salt tolerance by regulating some salt-tolerant genes in rice.

## 2. Results

### 2.1. Expression Pattern of OsWRKY54

**Figure 1:**
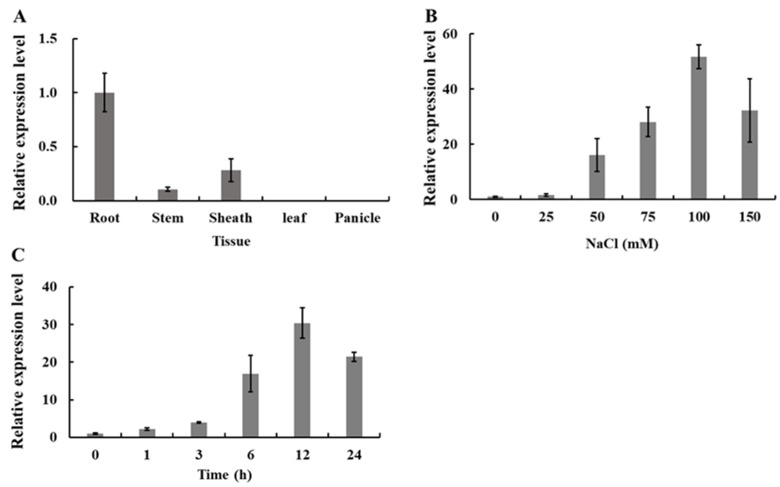
Expression profile of *OsWRKY54*. (**A**) The expression level of *OsWRKY54* in the roots, stems, leave, leaf sheaths, and panicles of rice plants at the heading stage. (**B**) The dose-dependent expression level of *OsWRKY54* in roots in response to NaCl treatment. Five-day-old rice seedlings were subjected to NaCl with different concentrations (0, 25, 50, 75, 100, and 150 mM) for 12 h. (**C**) The time-course expression level of *OsWRKY54* in rice roots in response to NaCl treatment. Five-day-old rice seedlings were treated with 100 mM NaCl at different times (0, 1, 3, 6, 12, and 24 h). *Histone H3* was used as an internal reference. Data represent mean ± SD (n = 4).

The expression of *OsWRKY54* in various rice organs, including spike, leaf blade, leaf sheath, stem, and root, was examined by quantitative RT-PCR analysis (Figure 1A). *OsWRKY54* was primarily expressed in the roots but not in the leaf sheath and panicle. Moreover, NaCl up-regulated its expression in the roots dose-dependently (Figure 1B). A time-course experiment showed that *OsWRKY54* expression was the highest at 12 h of salt treatment (Figure 1C). These results suggested that NaCl regulated *OsWRKY54*.

### 2.2. Subcellular Localization of OsWRKY54

**Figure 2:**
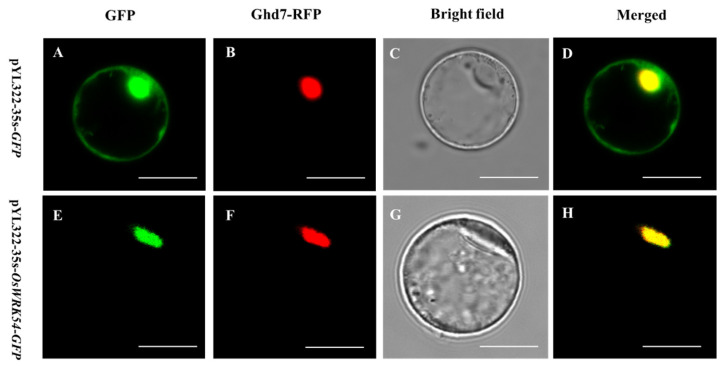
Subcellular localization of OsWRKY54. In rice leaf protoplast cells, OsWRKY54-GFP fusion or GFP alone was co-expressed with Ghd7-RFP, a nuclear marker. (**A**–**D**) show the localization of Ghd7-RFP and GFP. (**E**–**H**) show the localization of OsWRKY54-GFP and Ghd7-RFP. GFP, RFP, and merged RFP and GFP. Scale bar = 10 μm.

The subcellular localization of OsWRKY54 was determined by the transient expression of GFP-tagged OsWRKY54 in rice protoplast cells. *GFP* or an *OsWRKY54-GFP* fusion was transformed into rice protoplasts along with a nuclear marker *Ghd7-RFP* [16]. The green signal of OsWRKY54-GFP was primarily colocalized with the red signal of nuclear markers in rice protoplast cells. On the contrary, the signals of GFP alone were localized to both cytosol and nucleus, indicating that OsWRKY54 is a nuclear protein (Figure 2).

### 2.3. Cell-Specific Expression of OsWRKY54

**Figure 3:**
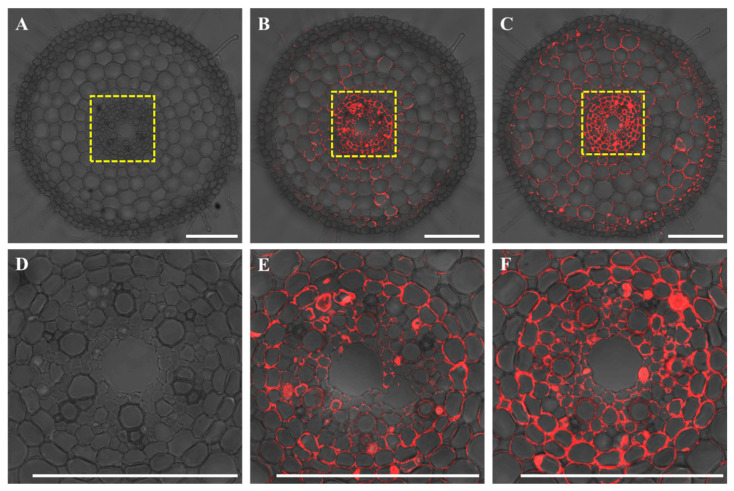
Tissue and cell specificity of *OsWRKY54* expression. The wild-type rice (**A**) and *ProOsWRKY54:GFP* transgenic rice (**B**,**C**) were exposed to 0 (**A**,**B**) or 100 mM NaCl (**C**) for 12 h. Roots were used for immunostaining. (**D**–**F**) represents the magnified image of the yellow boxed area in (**A**–**C**), respectively. Red shows the signal of the anti-GFP antibody. Scale bar = 100 μm.

The ProOsWRKY54-GFP transgenic rice was generated to examine the cellular specificity of OsWRKY54 expression in rice roots. Immunostaining of transgenic rice roots using an antibody against GFP showed that the OsWRKY54 promoter (red signal) was expressed in all the cell layers (Figure 3B). Exposure to NaCl enhanced the intensity of the signal (Figure 3C). Moreover, the signal in the stele was more potent than that in other cell layers under both normal and salt conditions (Figure 3E,F). Meanwhile, no GFP signal was detected in wild-type roots (Figure 3A,D).

### 2.4. Knockout of OsWRKY54 Decreased Salt Tolerance of Rice

**Figure 4:**
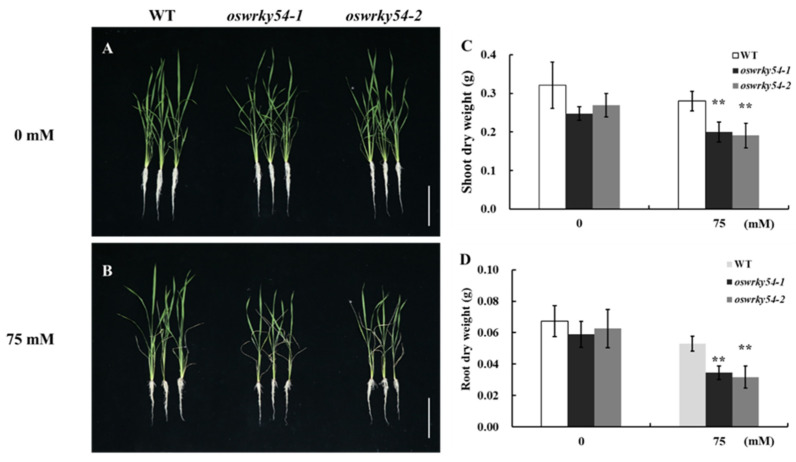
Knockout of *OsWRKY54* reduces rice salt-tolerance. (**A**,**B**) Growth phenotype of WT and two *oswrky54* mutant lines after treatment with 0 (**A**), 75 (**B**) mM NaCl for 14 days. Scale bar = 10 cm. (**C**,**D**) Dry weight of the roots and shoots in WT and two *oswrky54* mutant lines after treatment with 0 or 75 mM NaCl. Thirty-d-old rice seedlings were subjected to 0 or 75 mM NaCl for fourteen days. Data represent mean ± SD (n = 4). Asterisks denote a significant difference (** *p* < 0.01; Tukey’s test).

Given that the expression of *OsWRKY54* was induced by salt treatment, it was hy-pothesized that *OsWRKY54* might be involved in rice salt-tolerance. Thus, we generated two independent knockout mutant lines for the *OsWRKY54* gene by CRISPR-Cas9 technology: one with a C insertion (*oswrky54-1*) and the other with an A instertion (*oswrky54-2*), at different locations within the second exon (Figure S1). These insertions resulted in frame-shift mutations in *OsWRKY54*. Both knockout lines were used to compare salt tolerance with the wild-type rice (WT). Under normal conditions, the growth of two *oswrky54* mutant lines was similar as the WT (Figure 4A). However, post salt (75 mM NaCl) treatment, two knockout lines displayed more withered leaves than the WT (Figure 4B). The dry weight of the shoots and roots was also less than that of the WT (Figure 4C,D). These data indicated that mutating *OsWRKY54* increased the sensitivity of the rice plants to salt stress.

### 2.5. Loss of OsWRKY54 Alters Shoot Na^+^/K^+^ Homeostasis of Transgenic Rice

**Figure 5:**
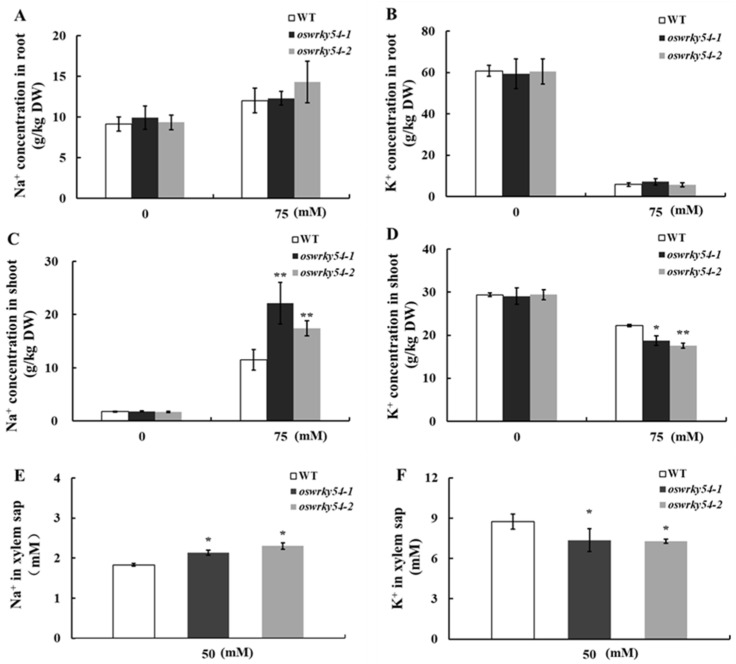
Na^+^ and K^+^ concentration in roots, shoots, and xylem sap of WT and *oswrky54* mutants under control conditions or salt treatment. (**A**) Na^+^ concentration in roots of the plants subjected to 0 or 75 mM NaCl for 14 d. (**B**) K^+^ concentration in roots of the plants subjected to 0 or 75 mM NaCl for 14 d. (**C**) Na^+^ concentration in shoots of the plants subjected to 0 or 75 mM NaCl for 14 d. (**D**) K^+^ concentration in shoots of the plants subjected to 0 or 75 mM NaCl for 14 d. (**E**) Na^+^ concentration in the xylem sap subjected to 50 mM NaCl for 6 h. (**F**) K^+^ concentration in the xylem sap of the plants subjected to 50 mM NaCl for 6 h. Asterisks denote a significant difference (* *p* < 0.05, ** *p* < 0.01; Tukey’s test). The data represent the mean ± SD (n = 4).

Next, Na^+^ and K^+^ concentration in WT and *oswrky54* mutant plants under normal and salt conditions was measured to test if *OsWRKY54* was involved in regulating tissue Na^+^/K^+^ homeostasis in rice. Under normal conditions, there were no apparent differences in the Na^+^ and K^+^ concentration of the roots and shoots between WT and *oswrky54* mutant lines (Figure 5A–D). After salt treatment with 75 mM NaCl, Na^+^ concentration in shoots of two knockout lines was about 2-fold higher than that of the WT plants. In contrast, K^+^ concentration in the mutant shoots was markedly lower (Figure 5C,D). Still, there was no significant difference in Na^+^ and K^+^ concentration in the roots between the WT and *oswrky54* mutant plants (Figure 5A,B). Furthermore, increased Na^+^ concentration and reduced K^+^ concentration were observed in the xylem sap of the *oswrky54* mutant lines relative to the WT (Figure 5E,F). These results suggested that OsWRKY54 affected the Na^+^/K^+^ homeostasis of rice shoot parts by regulating the root-to-shoot transport of Na^+^ and K^+^ under salt-stress conditions.

### 2.6. Transcriptional Activation Analysis of OsWRKY54

Since *OsWRKY54* is a rice WRKY TF family member, its transcriptional activation activity was examined in yeast. The full-length coding region of *OsWRKY54* was fused to the DNA-binding domain of GAL4, producing the pGBK-*OsWRKY54* construct. Yeast cells harboring the pGBK-*OsWRKY54* construct showed significant growth on selective growth medium compared to those carrying the empty vector pGBK (Figure S2). These results indicated that OsWRKY54 possessed self-activation activities in yeast.

### 2.7. OsWRKY54 Affects the Expression Profiles of Many Stress-Related Genes

**Figure 6:**
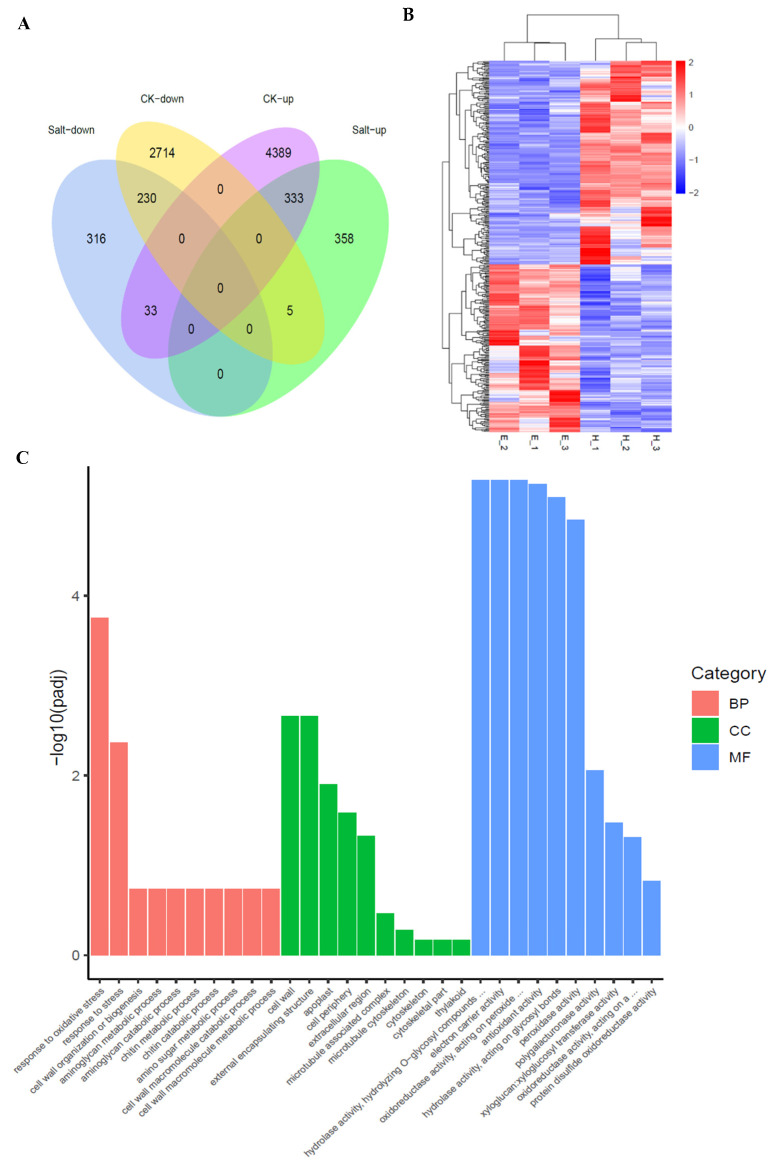
Transcriptome analysis of the *OsWRKY54*-regulated genes. (**A**) Venn diagram showing the *OsWRKY54*-regulated genes in the *oswrky54* mutant and WT. CK-up: up-regulated genes in the *oswrky54* mutant compared with WT under normal conditions (fold-change > 2.0). Salt-up: up-regulated genes in the *oswrky54* mutant compared with WT under salt stress (fold-change > 2.0). Salt-down: down-regulated genes in the *oswrky54* mutant compared with WT under salt stress (fold-change < 0.5). CK-down: down-regulated genes in the *oswrky54* mutant compared with WT under normal conditions (fold-change < 0.5). (**B**) Cluster analysis of the OsWRKY54-regulated genes under salinity conditions. E1-E3 are three biological replicates of wild-type plants, and H1-H3 are three biological replicates of the *oswrky54* plants. Red: up-regulated; blue: down-regulated. (**C**) GO enrichment description of down-regulated genes in the *oswrky54* mutant compared with WT under salt stress. MF (molecular function), CC (cellular component), and BP (biological process).

**Figure 7:**
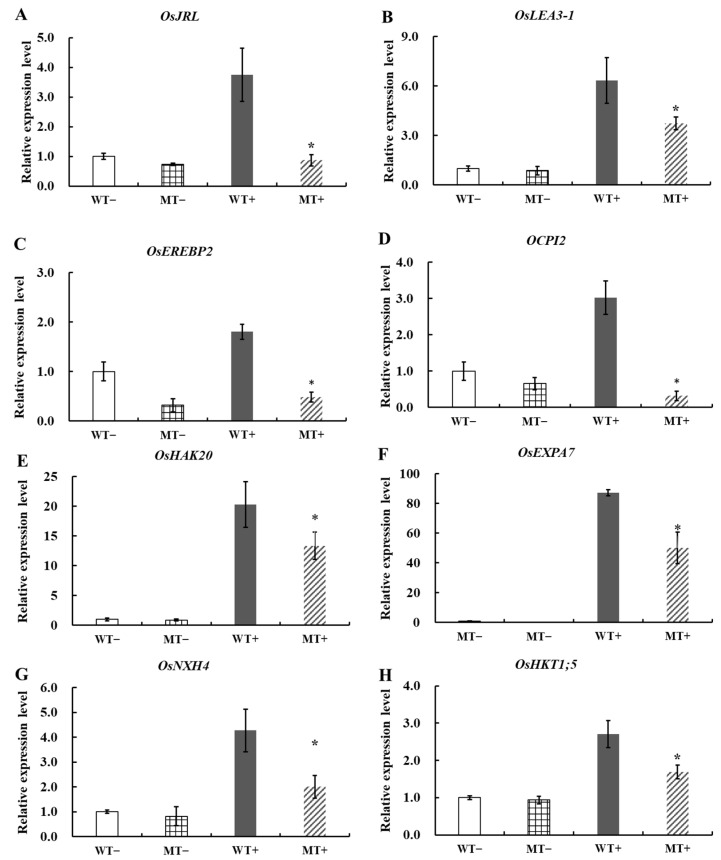
The relative expression level of candidate genes regulated by OsWRKY54 under control and high salinity conditions. *OsJRL* (**A**), *OsLEA3-1* (**B**), *OsEREBP2* (**C**)*, OCPI2* (**D**)*, OsHAK20* (**E**), *OsEXPA7* (**F**), *OsNXH4* (**G**), *OsHKT1;5* (**H**). WT− and MT− represent the wild-type (WT) and the *OsWRKY54* knockout mutant (MT) under control conditions, respectively. WT+ and MT+ represent the wild-type (WT) and the *OsWRKY54* knockout mutant (MT) treated with 100 mM NaCl for 12 h, respectively. The data indicate the means ± SD (n = 4), * *p* < 0.05.

The root transcriptome of the WT and a knockout mutant *oswrky54* (MT) under normal and salt conditions was analyzed by the RNA-seq approach to identify the *OsWRKY54*-regulated genes responsible for salt stress response. Based on the criterion of a two-fold change, 7704 and 1275 differentially expressed genes (DEGs) were identified between the mutant and WT lines under normal and salt conditions, respectively (Figure 6A; Table S1). Among them, there are 333 up-regulated DEGs and 230 down-regulated DEGs under both two conditions. The down-regulated genes in both normal and salt conditions were defined as the candidate genes regulated by OsWRKY54 (Figure 6A). We also found that some DEGs showed the opposite changes in expression levels between WT and MT under salt conditions (Figure 6B). QRT-PCR analysis was carried out to validate the reliability of the RNA-seq data. We selected eight genes that were reported to be involved in rice salt-tolerance, including *OsJRL* (Os01g0348900), *OsLEA3-1* (Os05g0542500), *OsEREBP2* (Os01g0868000), *OCPI2* (Os01g0615100), *OsHAK20* (Os02g0519100), *OsEXPA7* (Os03g0822000), *OsNHX4* (Os06g0318500), and *OsHKT1;5* (Os01g0307500) [17,18,19,20,21,22,23,24] (Figure 7). The qRT-PCR data showed that the expression of these genes in MT were significantly lower than those in WT under salt treatment, which was highly correlated with the transcriptome data. Additionally, a gene ontology (GO) enrichment analysis of down-regulated DEGs showed that most genes were mainly related to stress response (Figure 6C). These results indicated that *OsWRKY54* probably affected various stress-related genes in rice to regulate salt tolerance.

### 2.8. OsHKT1;5 Is a Direct Target of OsWRKY54

**Figure 8:**
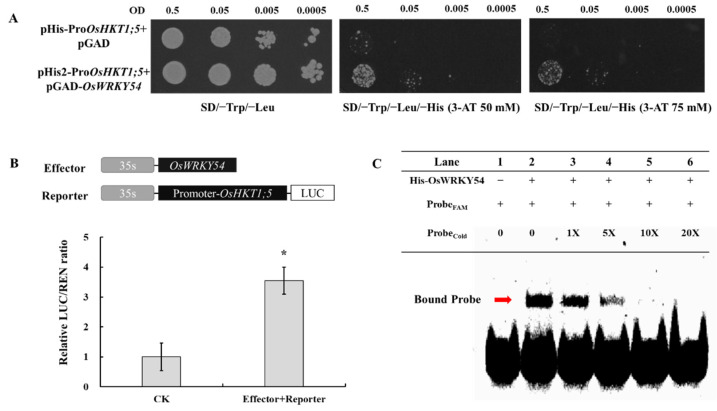
Interaction of OsWRKY54 with the promoter of *OsHTK1;5*. (**A**) OsWRKY54 binding to the *OsHKT1;5* promoters in yeast. Different pairs of plasmids (pGAD-OsWRKY54 and pHIS2-*ProOsHKT1;5*, or control pGADT7 and pHIS2) were transformed into yeast cells. Transformed cells were spotted on SD medium containing 0, 50, or 75 mM 3-amino-1,2,4-triazole at 30 °C for 3 days without His. (**B**) Activation of the OsHKT1;5 promoters by OsWRKY54 in rice protoplast. *ProOsHKT1;5:LUC* and 35S:OsWRKY54 were co-introduced into rice protoplast. The activity of REN gene was used as internal control. The relative ratio of LUC/REN is shown as mean ± SD (n = 3), * *p* < 0.05. (**C**) EMSA showing the binding of His-OsWRKY54 to the W-box element in the *OsHKT1;5* promoter. FAM-labeled probe (FAM) was shown in lanes 1–6. An unlabeled probe (Cold) was used in 0-, 1-, 5-, 10-, or 20-fold molar excess (lanes 2–6).

RNA-seq and qRT-PCR data showed that the expression level of *OsHKT1;5* in the *OsWRKY54* mutant roots is markedly lower than that in the wild-type roots under high-salinity conditions (Figure 7H; Table S1). A luciferase (LUC) assay in rice protoplasts was performed using CaMV35-driven *OsWRKY54* as the effector and the LUC as the reporter to determine whether *OsWRKY54* can activate the promoter of *OsHKT1;5*. LUC driven by the *OsHKT1;5* promoter (*ProOsHKT1;5*:*LUC*) was individually transformed or co-transformed with *Pro35S*: *OsWRKY54* in rice protoplasts. The value of LUC/REN in cells containing both *ProOsHKT1;5*:*LUC* and *OsWRKY54* was 3.5 times that in cells containing *ProOsHKT1;5:LUC* alone (Figure 8B), indicating that *OsWRKY54* activated the transcriptional activity of the *OsHKT1;5* promoters.

To further verify if *OsWRKY54* had the ability to bind to the *OsHKT1;5* promoter, we conducted a yeast one-hybrid (Y1H) assay. A 2.0 kb promoter fragment of *OsHKT1;5* was fused with the *HIS3* reporter gene, generating the pHIS-*ProOsHKT1;5* construct. Yeast cells containing pHIS-*ProOsHKT1;5* plus pGADT7-*OsWRKY54* grew better on these selective media than those containing pHIS-*ProOsHKT1;5* and the empty vector pGADT7 (Figure 8A), suggesting that OsWRKY54 could directly bind to the *OsHKT1;5* promoters.

Finally, electrophoresis mobility shift assays (EMSAs) were used to examine whether *OsWRKY54* is capable of binding specifically to the W-box element in the promoter of *OsHKT1;5*. Based on plantCARE (http://bioinformatics.psb.ugent.be/webtools/plantcare/html/, accessed on 1 November 2020) analysis, one probe covering the W-box motif (1482–1502 bp from the *OsHKT1;5* start codon) was designed. FAM labeling showed that OsWRKY54 could form the complex with the FAM-labeled W-box probe. Furthermore, increasing the concentration of the same unlabeled probe markedly inhibited the formation of an OsWRKY54/FAM-labeled probe band (Figure 8C). These results indicated that OsWRKY54 physically interacted with the *OsHKT1;5* promoter sequence via the W-box motif and activated its activity.

## 3. Discussion

The WRKY TFs were demonstrated to have essential roles in various abiotic stresses such as drought, salt, and chilling [9,10,11,12,13,14,25]. However, there have been limited reports on the involvement of WRKY TFs in rice salt-tolerance until now. Here, the function of a novel rice WRKY TF OsWRKY54 in salt stress was elucidated. *OsWRKY54* was predominantly expressed in roots and rapidly up-regulated by salt stress. Knocking out *OsWRKY54* by CRISPR-Cas9 reduced rice tolerance to salt stress. Moreover, under salt conditions, Na^+^ concentration in the shoot and transcript profiles of many stress-related genes in roots was changed in the *OsWRKY54* mutants compared to the WT. These indicated that *OsWRKY54* is an essential component of salt tolerance in rice.

Previous studies have reported that three major Na^+^ transporters (SOS1, NHX, and HKT) played critical roles in plant resistance to salt stress [26]. The SOS-mediated pathway was considered to transport Na^+^ out of the root cells, whereas most NHX transporters were proposed to be responsible for Na^+^ detoxification by compartmentalizing Na^+^ into the vacuoles. Additionally, HKT transporters are reported to play significant roles in limiting Na^+^ transport to the aerial part organs. Since the disruption of *OsWRKY54* resulted in increased Na^+^ concentration in the xylem and shoots, there was the possibility that OsWRKY54 was involved in maintaining the Na^+^ homeostasis of rice aerial parts under saline conditions by regulating the above-mentioned Na^+^ transporter genes. The RNA-seq- and qRT-PCR-based analysis demonstrated that the expression levels of *OsNHX4* and *OsHKT1;5* in the wild type were significantly higher than the *OsWRKY54* mutant under salt stress conditions. The down-regulation of *OsNHX4* in the mutants may lead to less Na sequestered into the vacuoles in roots, whereas the reduction in *OsHKT1;5* expression resulted in more Na^+^ transfer to the shoot. Furthermore, YIH, EMSA, and Dual-LUC transient expression assays confirmed that OsWRKY54 physically bound to the promoter sequences of *OsHKT1;5* via its W-box motif and promoted its expression. These results suggested that knocking out *OsWRKY54* directly or indirectly downregulated the expression of *OsNHX4* and *OsHKT1;5*, causing greater root-to-shoot translocation of Na^+^, and resulted in more sensitivity of rice to salt stress.

Furthermore, RNA-seq analysis showed 230 down-regulated DEGs in the *OsWRKY54* mutant roots compared with the WT under control and high-salt conditions. Go enrichment analysis demonstrated that most down-regulated DEGs were related to abiotic stress. The qRT-PCR study showed that a few of the DEGs implicated in rice salt-tolerance were regulated by OsWRKY54, including *OsJRL*, *OsLEA3-1*, *OsEREBP2*, *OCPI2*, *OsHAK20*, and *OsEXPA7*. *OsJRL* was a mannose-binding jacalin-related lectin gene in rice. The overexpression of *OsJRL* in rice enhanced salinity tolerance by increasing the expression of several stress-related genes [17]. *OsLEA3-1*, a late embryogenesis abundant protein, was reported to participate in drought, salt, and abscisic acid (ABA) responses. The *OsLEA3-1*-overexpression lines showed drought and high salt stress tolerance in rice [18]. *OsEREBP2* was a transcription factor of the AP2/ERF family and participated in rice salt-tolerance by negatively regulating the *OsRMC* expression, which was receptor-like kinase *OsRMC* [19]. *OCPI2* encoding a rice chymotrypsin protease inhibitor 2 interacted with a rice E3 ligase OsMAR1 and played an essential role under salinity stress [20]. *OsEXPA7*, an expansin gene, was involved in rice salt-tolerance by coordinating cell wall loosening, ion homeostasis, and ROS scavenging [21]. In rice, OsHAK20 belonging to the KT/HAK/KUP family functioned as a high-affinity K^+^ transporter and was involved in regulating K^+^ homeostasis [22]. These indicate that the involvement of OsWRKY54 in salt tolerance was attributed to modulating the expression of the genes mentioned above.

In addition, the phylogenetic analysis demonstrated that OsWRKY54 shared high identity with OsWRKY48 (53%), OsWRKY21 (40%), and OsWRKY70 (38%) at the amino acid level (Figure S3). *OsWRKY70* was involved in indole-and JA-mediated defense response in herbivore gnaw via interaction with *OsMPK3* and *OsMPK6* [27,28]. *OsWRKY21* was a potential positive regulator of chilling tolerance [25]. A recent study also reported that *OsWRKY21* interacted with *OsWRKY108* in the nucleus and jointly regulated the expression of *OsPHT1;1* to maintain Pi homeostasis in rice [29]. Additionally, *OsWRKY21* was found to modulate rice’s plant height and stem development [30]. These reports demonstrated that the homologous genes of *OsWRKY54* played essential roles in response to biotic and abiotic stress in rice. The transcriptome sequencing analysis revealed that the expression of many stress-related genes might be regulated by OsWRKY54, implying that, like its homologous genes, OsWRKY54 may also play a role in other stress responses.

Thus, OsWRKY54, a novel nuclear-localized WRKY transcription factor, participated in rice salt-tolerance by regulating some critical Na^+^ transport and stress-related genes expressed in roots.

## 4. Materials and Methods

### 4.1. Generation of OsWRKY54 Knockout Mutants

The knockout lines of *OsWRKY54* were created by the CRISPR/Cas9 genome-targeting technology based on a previously described method [31]. First, two target sequences of *OsWRKY54* were fused to the single-guide RNA (sgRNA) by overlapping PCR, forming *pU6a*-*OsWRKY54*-*sgRNA* or *pU6b*-*OsWRKY54*-s*gRNA* fragments. Then, using the *BsaI* restriction enzyme, these sgRNAs were assembled into the pYLCRISPR/Cas9Pubi vector. Final vectors containing sgRNAs were transformed into rice callus via *Agrobacteria tumefaciens stain* EHA105. The target sites’ fragments were amplified by PCR from the transgenic plants and directly sequenced to detect the homozygous mutant lines. The homozygous mutant lines were identified and used for further analysis. All the primers are listed in Table S2.

### 4.2. Plant Materials and Growth Conditions

The WT rice (cv. Nipponbare) and two CRISPR-Cas9 knockout lines of *OsWRKY54* were used in this work. Rice seeds were soaked in deionized water for two days, and then the germinated seeds were transferred to a net floating on a solution with 0.5 mM CaCl_2_. After five days, the rice seedlings were cultured in a half-strength Kimura B solution in a greenhouse.

Roots, stems, leaves, leaf sheaths, and panicles were sampled from WT plants at the heading stage for organ specificity analysis, and 5-day-old WT seedlings were used to analyze the response of *OsWRKY54* to salt stress in roots. The WT seedlings were exposed to NaCl solution with different concentrations (0, 25, 50, 75, 100, and 150 mM) for 12 h. The WT seedlings were treated with 100 mM NaCl for different times (0, 1, 3, 6, 12, and 24 h).

### 4.3. Gene Expression Analysis

Total RNA was extracted from various organs using the Trizol reagent kit (Invitrogen, Carlsbad, CA, USA). Next, genomic DNA removal and reverse transcription reactions were performed using Hiscript II Q RT SuperMix Kit (Vazyme, Nanjing, China). The qRT-PCR assay was performed on the Step One Plus Real-Time PCR System (Analytik Jena AG, Jena, Germany) using ChanQTM SYBR Color qPCR Master Mix (Vazyme, Nanjing, China). *Histone H3* was used as an internal reference for normalization. The formula 2^−ΔΔCT^ calculated the relative expression levels of the genes. All the primers are indicated in Table S2.

### 4.4. Subcellular Localization of OsWRKY54

The full-length coding sequence of *OsWRKY54* was cloned in frame after the GFP in the pYL322-GFP vector to examine the subcellular localization of OsWRKY54. The empty vector pYL322*-GFP* or pYL322*-GFP-OsWRKY54* was introduced into rice protoplasts along with a nucleus marker (RFP-OsGhd7) using a previously described method [26]. After 12–16 h cultivation, the protoplast cells were placed under a confocal laser scanning microscope, and the GFP fluorescence signal was detected and photographed (TCS SP8; Leica, Weztlar, Germany).

### 4.5. Tissue Localization of OsWRKY54

A 2036 bp promoter (upstream region of the transcription start site) of *OsWRKY54* was obtained from Nipponbare genomic DNA by PCR. The promoter fragment was cloned in front of the GFP in pCAMBIA1300-*GFP* vector to make a construct carrying the *OsWRKY54* promoter-*GFP* fusion. The resulting construct (*ProOsWRKY54:GFP*) was transformed into Nipponbare.

The seedling (5-d-old) of WT and *ProOsWRKY54:GFP* transgenic lines prepared as described above were treated with 0 or 100 mM NaCl for 12 h. The roots were sampled for immunostaining with the GFP antibody as described by Yamaji and Ma [32] to determine the tissue-specificity localization of OsWRKY54 in roots. The fluorescent signal was detected by the confocal laser scanning microscope (TCS SP8, Leica, Weztlar, Germany).

### 4.6. Physiological Characterization of OsWRKY54 Mutant Lines

The seedling (5-d-old) of WT and two *OsWRKY54* mutant lines prepared as described above were grown 1/2 Kimura B nutrition solution for 25 d and then transferred to a nutrient solution containing 0 or 75 mM NaCl for another 14 days. After the treatment, plants were photographed. Finally, the shoots and roots of the WT and *OsWRKY54* mutant lines were separately sampled for the dry weight and elemental measurement.

### 4.7. Na/K Concentration in Xylem Sap

The seedling (5-d-old) of WT and two *OsWRKY54* mutant lines prepared as described above were grown in 1/2 Kimura B nutrition solution for 25 d and then transferred to a solution supplemented with 50 mM NaCl for 6 h. After the treatment, the seedling shoots (2 cm above the roots) were cut with a sharp blade, and the sap was collected from the cut surface for 1 h. The Na/K concentration of the xylem sap was measured as described below.

### 4.8. Determination of Na/K Concentration

All the dried samples were digested with 65% HNO_3_. The Na and K concentrations in the xylem sap and digested solution were measured using ICP-MS (Plasma Quant MS; Analytik Jena AG, Jena, Germany).

### 4.9. RNA-Seq Assay

The 18-day-old seedlings of WT and the *oswrky54* mutant line (MT) were cultured in a solution containing 100 mM NaCl for 24 h. The roots of all lines were used for total RNA extraction, cDNA synthesis and purification. The cDNA library was sequenced as 150 bp paired-end reads using the IlluminaHiSeq system, and 89.3–94.3% of the reads were mapped to the rice reference genome. Deseq2 was applied for differential gene expression analysis. Genes with |log_2_-ratio| > 1 and *p*-value < 0.05 under salt condition were identified as DEGs (differentially expressed genes). GO (gene ontology; http://geneontology.org/, accessed on 1 October 2020) enrichment analysis of DEGs was based on hypergeometric tests. The RNA-seq raw data of all samples were deposited in the Sequence Read Archive at the National Center for Biotechnology Information (http://www.ncbi.nlm.nih.gov/sra, accessed on 3 October 2022) under accession number SRP400051.

### 4.10. Dual-LUC Transient Expression Assay

The promoter sequence (2036-bp from the start codon) of *OsWRKY54* was cloned in front of the firefly *LUC* gene in pGreen II 0800-LUC upstream, generating the reporter *ProOsWRKY54:LUC* construct. The full-length coding sequence of the *OsWRKY54* gene was cloned after the CaMV 35S promoter in the pGreen II 62-SK vector, forming the effector vector. As an internal control, the CaMV 35S promoter drove the reporter vector’s Renilla LUC (REN) gene. As previously described, the reporter and effector vectors were transformed into rice protoplasts [15]. The activity of LUC and REN was detected by a Dual-LUC Reporter Assay System (Promega, Madison, WI, USA). The primers were listed in Supplemental Table S2.

### 4.11. Yeast Assays

The full-length coding sequence of *OsWRKY54* was amplified by PCR and introduced by ligation into the pGBKT7 vector, generating the pGBKT7-*OsWRKY54* construct to investigate the transactivation activity of *OsWRKY54*. The empty vector pGADT7 plus the pGBKT7 or pGBKT7-*OsWRKY54* construct was co-introduced into AH109 yeast cells. The yeast colonies were grown on the plates containing SD/Trp- or SD/Trp-/His-/Ade- medium and incubated at 30 °C.

A yeast one-hybridization assay was employed to determine the binding ability of OsWRKY54 to the *OsHKT1;5* promoter. A 2036-bp promoter sequence of *OsHKT1;5* was cloned in front of the *HIS3* reporter gene in pHIS2 vector (Clontech, Shiga, Japan), generating the pHIS-*ProOsHKT1;5* construct. The full-length OsWRKY54 was cloned in-frame after the GAL4 activation domain in pGADT7 (Clontech) to produce the pGAD-*OsWRKY54* construct. A pair of these plasmids (pGAD-*OsWRKY54* and pHIS2-*ProOsHKT1;5*, or control pGADT7 and pHIS2) was transformed into yeast strain AH109. The yeast colonies were spotted on SD/-Leu/-Trp and SD/-Leu/-Trp/-His medium containing 0–75 mM 3-amino-1,2,4-triazole and cultured at 30 °C for 3 days. The primers used for constructing the vectors are listed in Supplemental Table S2.

### 4.12. EMSA

For EMSA, the full-length ORF of *OsWRKY54* was introduced by ligation into the pRSFDuet-1 vector to form *OsWRKY54-His* fusion, producing the pRSFDuetI-His-*OsWRKY54* construct. The resulting construct was expressed in *Escherichia coli* (BL21). Following the instructions, the recombinant fusion protein His-OsWRKY54 was purified with Ni Sepharose 6 Fast Flow columns (GE). DNA oligonucleotides probe containing the W-box motif were synthesized with the forward strand labeled by 5′-carboxyfluorescein (FAM). The unlabeled probe with the same sequence was used as competitors. EMSA was performed as previously described [33]. The probe sequences for EMSA are indicated in Supplemental Table S2.

## Data Availability

All of the data supporting the conclusions of this article are provided within the article and in its additional files. All of the data and materials are available upon reasonable request from the corresponding author.

## Supplementary Materials

The following supporting information can be downloaded at https://www.mdpi.com/article/10.3390/ijms231911999/s1.
