# Supplementary material for: The WRKY Transcription Factor OsWRKY54 Is Involved in Salt Tolerance in Rice
Source: Int J Mol Sci. 2022 Oct 9;23(19):11999. doi: 10.3390/ijms231911999 (PMC9569829; doi:10.3390/ijms231911999)
Supplement: Supplementary file 1 [file ijms-23-11999-s001.zip › Supplemental Figure S1-S3.pdf]

## Supplementary Figures

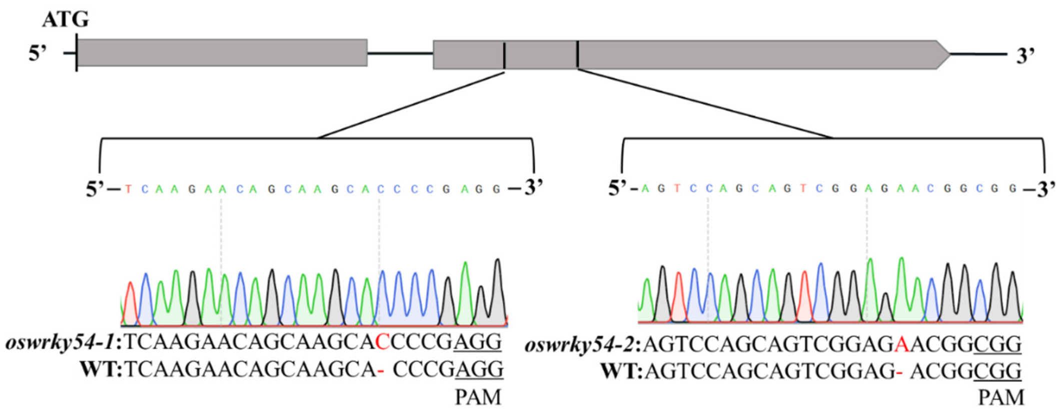


**Figure S1.** *OsWRKY54* sequence of two independent mutants generated by CRISPR/Cas9 mutagenesis. The grey box indicates the exon.

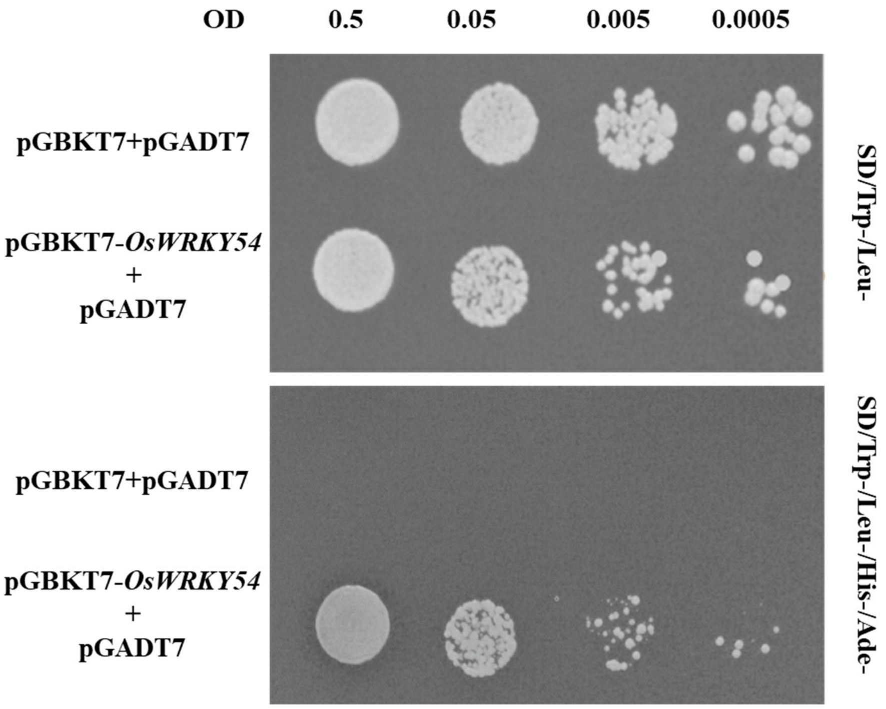


**Figure S2. Self-activation analysis of OsWRKY54.** Yeast carrying pGBKT7 (negative control) or pGBKT7-OsWRKY54 were spotted on the SD/Trp- or SD/Trp-/His-/Ade- plates for 3 days at 28 ℃.

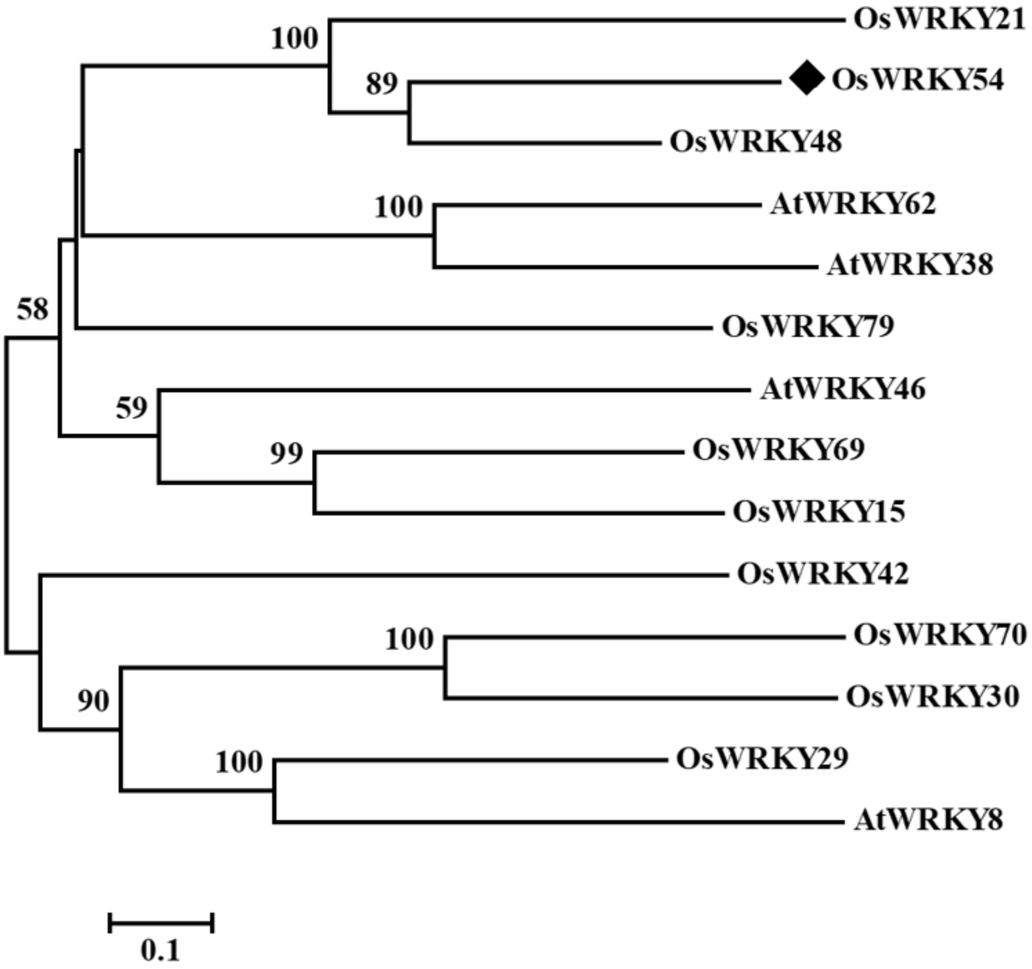


**Figure S3. Phylogenetic tree analysis of OsWRKY54.** Homologs sharing > 30% identity to OsWRKY54 were used to construct the phylogenetic tree (NCBI). Multiple sequence alignment was performed using the MEGA 7.0, and the phylogenic tree was created.
